# Inhibition of O-GlcNAcase leads to elevation of O-GlcNAc tau and reduction of tauopathy and cerebrospinal fluid tau in rTg4510 mice

**DOI:** 10.1186/s13024-017-0181-0

**Published:** 2017-05-18

**Authors:** Nicholas B. Hastings, Xiaohai Wang, Lixin Song, Brent D. Butts, Diane Grotz, Richard Hargreaves, J. Fred Hess, Kwok-Lam Karen Hong, Cathy Ruey-Ruey Huang, Lynn Hyde, Maureen Laverty, Julie Lee, Diane Levitan, Sherry X. Lu, Maureen Maguire, Veeravan Mahadomrongkul, Ernest J. McEachern, Xuesong Ouyang, Thomas W. Rosahl, Harold Selnick, Michaela Stanton, Giuseppe Terracina, David J. Vocadlo, Ganfeng Wang, Joseph L. Duffy, Eric M. Parker, Lili Zhang

**Affiliations:** 1 Department of Neuroscience, Kenilworth, NJ USA; 2 Department of In Vivo Pharmacology, West Point, PA USA; 3 Department of Molecular Biomarkers, Kenilworth, NJ USA; 4 Department of Pharmacokinetics, Pharmacodynamics and Drug Metabolism, Kenilworth, NJ USA; 5 Department of Neuroscience, West Point, PA USA; 6 Alectos Therapeutics Inc., Burnaby, BC Canada; 7 Department of In Vivo Pharmacology, Kenilworth, NJ USA; 8 Discovery Chemistry, Merck Research Laboratories, West Point, PA USA; 9 Discovery Chemistry Merck Research Laboratories, Kenilworth, NJ USA

**Keywords:** Tau, OGA, O-GlcNAc, Alzheimer’s disease, Tauopathy, Neurodegeneration

## Abstract

**Background:**

Hyperphosphorylation of microtubule-associated protein tau is a distinct feature of neurofibrillary tangles (NFTs) that are the hallmark of neurodegenerative tauopathies. O-GlcNAcylation is a lesser known post-translational modification of tau that involves the addition of N-acetylglucosamine onto serine and threonine residues. Inhibition of O-GlcNAcase (OGA), the enzyme responsible for the removal of O-GlcNAc modification, has been shown to reduce tau pathology in several transgenic models. Clarifying the underlying mechanism by which OGA inhibition leads to the reduction of pathological tau and identifying translatable measures to guide human dosing and efficacy determination would significantly facilitate the clinical development of OGA inhibitors for the treatment of tauopathies.

**Methods:**

Genetic and pharmacological approaches are used to evaluate the pharmacodynamic response of OGA inhibition. A panel of quantitative biochemical assays is established to assess the effect of OGA inhibition on pathological tau reduction. A “click” chemistry labeling method is developed for the detection of O-GlcNAcylated tau.

**Results:**

Substantial (>80%) OGA inhibition is required to observe a measurable increase in O-GlcNAcylated proteins in the brain. Sustained and substantial OGA inhibition via chronic treatment with Thiamet G leads to a significant reduction of aggregated tau and several phosphorylated tau species in the insoluble fraction of rTg4510 mouse brain and total tau in cerebrospinal fluid (CSF). O-GlcNAcylated tau is elevated by Thiamet G treatment and is found primarily in the soluble 55 kD tau species, but not in the insoluble 64 kD tau species thought as the pathological entity.

**Conclusion:**

The present study demonstrates that chronic inhibition of OGA reduces pathological tau in the brain and total tau in the CSF of rTg4510 mice, most likely by directly increasing O-GlcNAcylation of tau and thereby maintaining tau in the soluble, non-toxic form by reducing tau aggregation and the accompanying panoply of deleterious post-translational modifications. These results clarify some conflicting observations regarding the effects and mechanism of OGA inhibition on tau pathology, provide pharmacodynamic tools to guide human dosing and identify CSF total tau as a potential translational biomarker. Therefore, this study provides additional support to develop OGA inhibitors as a treatment for Alzheimer’s disease and other neurodegenerative tauopathies.

**Electronic supplementary material:**

The online version of this article (doi:10.1186/s13024-017-0181-0) contains supplementary material, which is available to authorized users.

## Background

Intra-neuronal accumulation of neurofibrillary tangles (NFTs) is one of the major pathological hallmarks of Alzheimer’s disease. The main component of NFTs is tau, a microtubule-binding protein that becomes hyperphosphorylated and aggregates into paired helical filaments during disease development [1]. Tau pathology, or tauopathy, is also present in a number of other neurodegenerative diseases, including progressive supranuclear palsy (PSP), corticobasal degeneration, frontotemporal dementia and Pick’s disease [2]. Dominant tau mutations have been identified that are associated with aggressive tauopathies, including frontotemporal dementia with Parkinsonism on chromosome 17 and PSP [3, 4]. These findings support a pathogenic role of tau in neurodegeneration and lead to the therapeutic hypothesis that reduction of tau pathology may be a viable approach to slow down the progression of diseases involving tauopathy.

Protein O-GlcNAcylation is a reversible post-translational modification involving addition of a single N-acetylglucosamine (O-GlcNAc) moiety onto the hydroxyl group of serine and threonine residues (reviewed by [5]). This modification is regulated by two enzymes in mammalian cells. O-GlcNAc transferase (OGT, EC 3.2.1.255) catalyzes the addition of O-GlcNAc to protein substrates and O-GlcNAcase (OGA, EC 3.2.1.169) catalyzes the hydrolytic removal of O-GlcNAc from proteins. Many cytoplasmic and nuclear proteins are subject to O-GlcNAcylation, and because this modification occurs on serine and threonine residues, it can potentially modulate protein phosphorylation directly on the same sites or indirectly on proximal sites [5].

The possible dynamic interplay between protein O-GlcNAcylation and phosphorylation has led to the hypothesis that inhibition of the OGA enzyme would promote O-GlcNAcylation of tau, thereby attenuating tau hyperphosphorylation and providing therapeutic benefit for AD and other tauopathies [6]. In fact, several studies in which the potent and selective OGA inhibitor Thiamet G was administered to transgenic mice overexpressing human tau have demonstrated positive effects on tau pathology, tau phosphorylation (p-tau) and/or behaviors and phenotypes thought to be dependent on tau pathology ([7–9], also reviewed by [6]). Although these findings generally support OGA inhibition as a promising approach to regulate tau pathology, there are significant discrepancies regarding whether and by what mechanism OGA inhibition affects tau aggregation, phosphorylation and/or O-GlcNAcylation. These discrepancies have hindered the progression of OGA inhibitors into human clinical development [10]. In addition, information and tools to guide human dosing of OGA inhibitors are very limited. The degree of OGA inhibition required to reduce tau pathology is unknown, and there are no translational biomarkers or sensitive, quantitative, high throughput assays that measure OGA inhibition and its effect on tau pathology in humans. Clarifying the mechanism by which OGA inhibition reduces tau pathology and addressing these translational gaps will greatly facilitate further advancement of this target for the treatment of tauopathies.

To further establish the mechanism of OGA inhibitors as a potential treatment for tauopathies and to develop transitional tools, we undertook a systematic assessment of the effects of the OGA inhibitor Thiamet G in rTg4510 mice. This mouse model of tauopathy has several features that facilitate the evaluation of potential therapeutic treatments [11–14]. We utilized a panel of previously developed high throughput, quantitative assays for various species of tau [13], which enabled us to detect pathological tau development at an early age in rTg4510 mice and to conduct well-powered studies to mitigate the high inter-animal variability inherent in this model. In addition, several novel assays were developed to measure O-GlcNAcylation of proteins in the brain, including an assay to directly measure O-GlcNAcylation of tau. These studies provide the first description of the pharmacology of OGA inhibition required for reduction of tauopathy, identify decreases in CSF tau as a translatable biomarker of pathological tau reduction by OGA inhibition, and confirm direct O-GlcNAcylation of tau as the likely mechanism by which OGA inhibition reduces pathological tau. Taken together, these studies pave a path forward for further development of OGA inhibitors to treat tauopathy in humans.

## Results

### Effect of OGA inhibition on O-protein levels

**Fig. 1:**
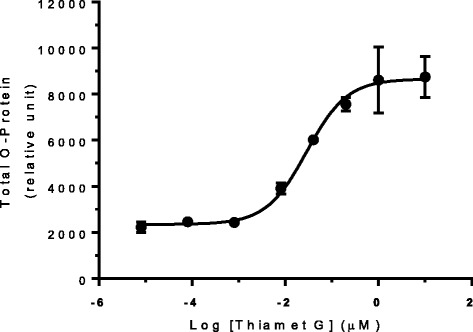
Dose-dependent increase of total O-protein in HEK293 cells treated with Thiamet G. A quantitative sandwich immunoassay (see Methods) was used to detect O-protein after treating the cells with various concentrations of Thiamet G for 6 h. The data shown are the mean ± standard error of the mean (SEM) from a single experiment performed in triplicate and are representative of three independent experiments

A sensitive, quantitative, high throughput MesoScale Diagnostics (MSD) sandwich immunoassay was developed to examine the effect of OGA inhibition on global protein O-GlcNAcylation (hereafter referred to as O-protein). This assay was validated using HEK293 cells treated with the potent and selective OGA inhibitor Thiamet G [15]. In these experiments, Thiamet G treatment led to a dose-dependent increase of O-protein, with an EC_50_ of 32 nM and a maximal O-protein elevation of 5- to 6-fold over levels observed in vehicle-treated cells (Fig. 1). The EC_50_ value and maximal O-protein elevation are consistent with the values previously reported after Thiamet G treatment of PC12 cells [15], but the quantitative precision, assay window and throughput of our MSD assay are significantly greater than the densitometric quantitation of Western blots used in previous studies. These advantages of the MSD assay permitted much more facile and accurate analysis of large numbers of samples from the subsequent animal studies.

**Fig. 2:**
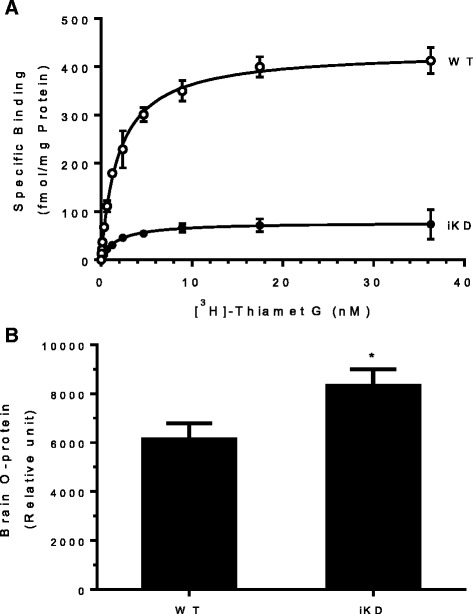
Determination of OGA enzyme and O-protein levels in OGA iKD mice. **a** Binding of [^3^H]Thiamet G in brain homogenates prepared from WT (*n* = 5) and OGA iKD (*n* = 4) mice was determined as described in Methods. Individual saturation binding curves were performed using total brain homogenates prepared from each animal and the data shown are the mean ± standard deviation of values for each group of animals. The K_D_ of [^3^H]Thiamet G binding to OGA was similar in WT and OGA iKD mouse brain (K_D_ = 1.7 nM and 1.9 nM, respectively), while the B_max_ values were reduced by 80% in the OGA iKD mouse relative to the WT mouse (433 fmol/mg protein and 78 fmol/mg protein, respectively). **b** Total O-protein in brain homogenates from WT (*n* = 5) and OGA iKD mice (*n* = 5) was measured by a quantitative sandwich immunoassay as described in Methods. Brain O-protein levels were 1.4-fold higher in OGA iKD mice relative to the WT mice. The data shown are the mean ± SEM. Each sample was assayed in duplicate. *p = 0.048 (t-test)

This assay was used in combination with genetic and pharmacological approaches to determine the dose of Thiamet G and the consequent degree of inhibition of OGA required for a therapeutic effect. Since constitutive knockout of the OGA gene is perinatal lethal [16], we developed an inducible OGA knockdown (OGA iKD) mouse model to study the effect of reduction of OGA enzymatic activity on brain O-protein levels (Additional file 1). After shRNA-mediated OGA knockdown for 10 days, reduction of OGA mRNA expression was observed across all tissues examined, with an average reduction of 70–80% (Additional file 2). Binding of [^3^H]Thiamet G in brain homogenates revealed an 80% reduction of OGA enzyme levels in OGA iKD mice compared to wild type mice (Fig. 2a), consistent with the magnitude of OGA mRNA reduction. Despite the substantial reduction of OGA mRNA and enzyme levels, the OGA iKD mice were normal with no overt phenotype. Interestingly, only a modest 1.4-fold increase of total O-protein was observed in the brains of OGA iKD mice despite the 80% reduction of OGA enzyme levels (Fig. 2b).

**Fig. 3:**
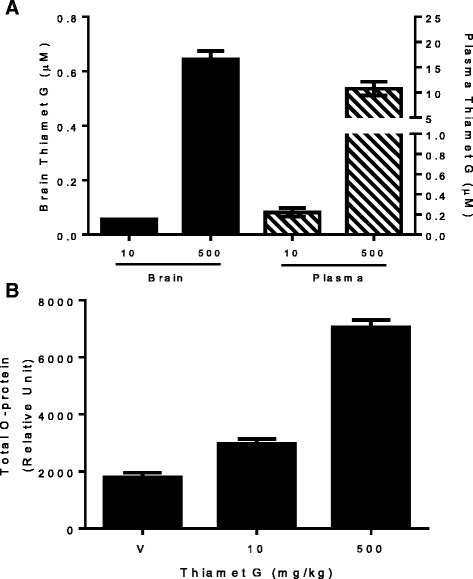
Brain O-protein levels following acute Thiamet G treatment. C57BL6 mice (*n* = 8 per group) were given a single oral dose of vehicle (water) or Thiamet G at 10 or 500 mg/kg. Brain and plasma were collected 6 h after dosing. **a** Concentrations of Thiamet G in plasma (*right*) and brain (*left*). **b** Elevation of O-protein in total brain homogenates after Thiamet G treatment. All data shown are the mean ± SEM. Each sample was assayed in duplicate

Pharmacological modulation of OGA activity was examined by treating mice with a single oral dose of either 10 or 500 mg/kg Thiamet G. Pharmacokinetic analysis showed a dose-dependent increase of Thiamet G in plasma and brain (Fig. 3a), although the compound has relatively poor brain penetration (brain to plasma ratio < 0.1). To determine the extent of brain OGA inhibition at these doses, we measured changes in O-protein levels in the brain. Administration of Thiamet G at 10 and 500 mg/kg led to a 1.7- and 4-fold increase of brain total O-protein, respectively (Fig. 3b). These data, along with the modest 1.4-fold increase of brain O-protein observed after 80% knockdown of brain OGA levels, suggest that a 500 mg/kg dose of Thiamet G is needed to significantly increase O-GlcNAc modification of brain proteins. Therefore, this dose was used in the subsequent chronic study.

### Effect of OGA inhibition on tauopathy in rTg4510 mice

**Fig. 4:**
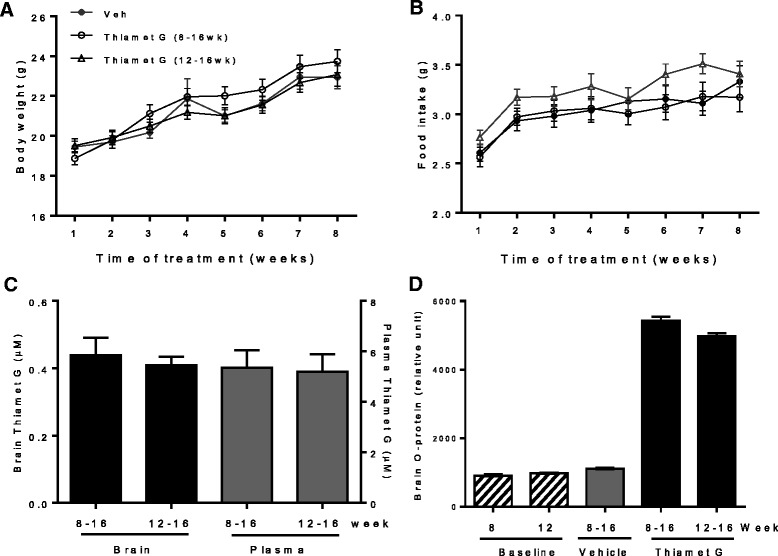
Effect of chronic treatment of rTg4510 mice with Thiamet G on body weight, food intake and brain O-protein. rTg4510 mice (*n* = 35 per group) were treated with Thiamet G (500 mg/kg/day in the diet) either for 8 weeks from 8 to 16 weeks of age or for 4 weeks from 12 to 16 weeks of age. Body weight and food consumption were monitored weekly. Plasma and brains were collected at the end of the study. Baseline groups of naïve (untreated) rTg4510 mice were also sacrificed at 8 or 12 weeks of age (*n* = 10 per age group). **a** Body weight and **b** food intake as measured weekly during the 8 weeks of the study. Animals in all groups showed an increase in body weight and food intake during the treatment period (*p* < 0.0001), but no significant differences were observed between the treatment groups (body weight, *p* = 0.6, food intake, *p* = 0.22). **c** and **d** Plasma and brain Thiamet G concentrations (**c**) and O-protein levels in total brain homogenates (**d**) measured at the end of the study. The data shown are the mean ± SEM. Each sample was assayed in duplicate

The rTg4510 mouse model uses the Ca^2+^-calmodulin kinase II promoter to overexpress human 0N4R tau carrying the P301L mutation [11]. This model recapitulates several important features of tau pathology observed in the brains of AD patients, including distribution in forebrain regions, multiple post-translational modifications of tau and elevated CSF tau [11, 13]. However, this model is also characterized by high inter-animal variability, an issue that may have contributed to variable OGA inhibitor effects in this model. Therefore, we mitigated this issue by using only female animals [13] and adequately powering the study to detect a relatively modest 30% effect of chronic treatment with 500 mg/kg/day Thiamet G on tauopathy in rTg4510 mice (see Methods). Two treatment durations of 4 weeks (from 12 to 16 weeks of age) or for 8 weeks (from 8 to 16 weeks of age) were studied to explore the time frame required to observe effects of OGA inhibition on tau pathology in the rTg4510 model. These ages and durations of treatment were chosen because they represent the early to middle stages of tauopathy in the rTg4510 model and thus correspond to the stages at which treatment is most likely to be effective, both in this model and in humans. The compound was well tolerated, and no overt adverse effects were observed in any treatment group. Animals in all groups gained weight as expected and there were no significant differences in food intake or body weight between treatment groups (Fig. 4a and b). Pharmacokinetic and pharmacodynamic analysis showed comparable terminal drug concentration in plasma and brain (Fig. 4c) and comparable elevation of brain total O-protein (Fig. 4d) in both Thiamet G-treated groups.

**Fig. 5:**
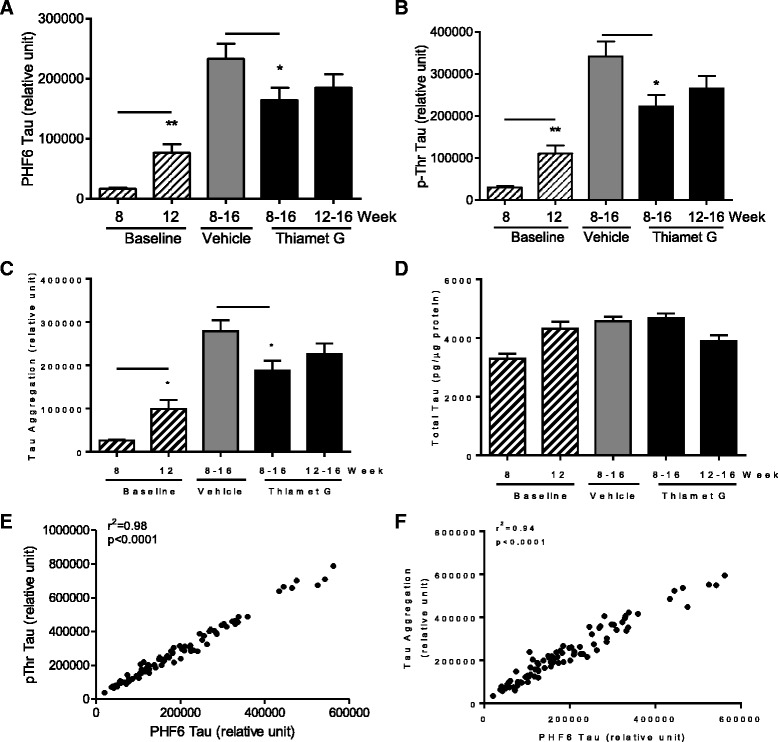
Chronic Thiamet G treatment reduces tauopathy in rTg4510 mice. Thiamet G (500 mg/kg/day) or vehicle were administered in diet to rTg4510 mice (*n* = 35 per group) from either 8–16 or 12–16 weeks of age. Baseline animals (*n* = 10 per group) were sacrificed at the indicated age and did not receive any treatment. Total tau, aggregated tau and various p-tau species were measured in the brain insoluble fraction using AlphaLISA-based immunoassays as described in Methods. The species of tau measured were: **a** p-tau recognized by PHF6; **b** global pThr phosphorylated tau; **c** tau aggregates; and **d** total tau. The data shown are the mean ± SEM. Each sample was assayed in duplicate. *p < 0.05 compared to vehicle-treated animals (t-test). **e** and **f** Statistically significant correlations between levels of pThr tau, PHF6 tau and tau aggregates were observed using data from all vehicle and Thiamet G-treated animals (r^2^ = 0.98 for pThr tau and PHF6 tau, r^2^ = 0.94 for tau aggregates and PHF6 tau, *p* < 0.0001 in both cases)

The effect of chronic Thiamet G treatment on tauopathy in rTg4510 mice was assessed by measuring various tau species in the insoluble fraction of brain homogenates. Dramatic increases of phosphorylated tau (recognized by anti-phosphothreonine (anti-pThr) and PHF6 antibodies) and aggregated tau were observed as the mice aged from 8 to 16 weeks (Fig. 5a-c; compare the increase in baseline levels of these tau species between 8 and 12 weeks of age and the further increase in the vehicle-treated group at 16 weeks of age). Insoluble species of hyperphosphorylated, aggregated tau are characteristic of tauopathy [2, 17], and these data thus indicate marked disease progression in the rTg4510 model during this period [13]. Compared to vehicle-treated rTg4510 mice, treatment with 500 mg/kg/day of Thiamet G for 8 weeks (from 8 to 16 weeks of age) led to a significant reduction of accumulated insoluble tau aggregates and pathological hyperphosphorylated tau species recognized by PHF6 and anti-pThr. Thiamet G treatment for 4 weeks (from 12 to 16 weeks of age) also led to a reduction of these pathological tau species, although the reduction did not reach statistical significance (Fig. 5a-c). Hyperphosphorylated tau species recognized by antibodies AT8 (pS202/pT205), AT270 (pT181) and AT180 (pT231) also showed a reduction in the brain insoluble fraction after Thiamet G treatment similar to that observed for species recognized by PHF6 and pThr (data not shown). In addition, a close correlation between different pathological tau species was observed despite large variations in each treatment cohort (Fig. 5e and f). There was no significant change in total tau in the insoluble fraction between 8 and 16 weeks of age, nor were total tau levels affected by Thiamet G treatment (Fig. 5d).

**Fig. 6:**
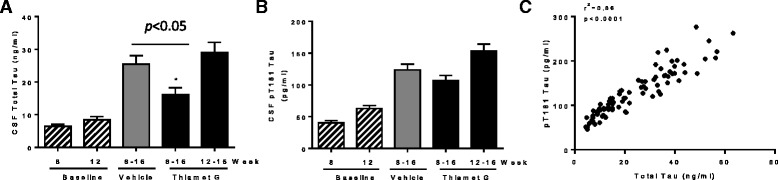
Effects of chronic Thiamet G treatment of rTg4510 mice on CSF tau. Thiamet G (500 mg/kg/day) or vehicle were administered in diet to rTg4510 mice (*n* = 35 per group) from either 8–16 or 12–16 weeks of age. Baseline animals (*n* = 10 per group) were sacrificed at the indicated age and did not receive any treatment. Eight weeks of treatment with Thiamet G treatment significantly reduced CSF total tau (**a**; *p < 0.05 by t-test) and CSF pT181 tau (**b**), although the latter effect did not reach statistical significance. CSF total tau and pT181 tau were measured as described in Methods. The data shown are the mean ± SEM. Each sample was assayed in duplicate. **c** Statistically significant correlation of CSF total tau and pT181 tau in vehicle and Thiamet G-treated groups (r^2^ = 0.86, *p* < 0.0001)

As previously reported [13], CSF total tau in 16 week old rTg4510 mice was elevated above the baseline levels in 8 and 12 week old mice (Fig. 6a), again indicating significant progression of tauopathy in this time frame. The rise of CSF total tau was reduced significantly after treatment of rTg4510 mice with 500 mg/kg/day of Thiamet G for 8 weeks from 8 to 16 weeks of age, but no statistically significant change was observed after treatment for 4 weeks from 12 to 16 weeks of age (Fig. 6a). Similar effects of Thiamet G treatment on CSF pT181 tau were observed, although the reduction of CSF pT181 tau after 8 weeks of treatment did not reach statistical significance (Fig. 6b). Once again, the change in CSF pT181 tau showed strong correlation with that of total tau (Fig. 6c). Overall, the effect of Thiamet G on the elevated CSF total tau and pT181 tau in rTg4510 mice closely mirrored the effects of the compound on various tau species in the insoluble fraction of brain (cf. Figs. 5 and 6).

### Detection of O-GlcNAc-tau (O-tau) after Thiamet G treatment

**Fig. 7:**
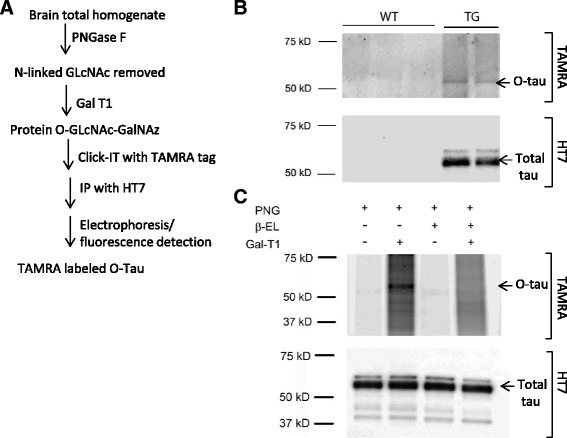
Validation of the “click” chemistry method for detection of O-tau. **a** Flow chart describing the procedure for O-tau detection (see Methods for further details). **b** Detection of a 55 kDa TAMRA-labeled protein in rTg4510 mouse brain homogenate (*upper panel*) that is recognized by the HT7 anti-tau antibody (lower panel). Note that this putative human O-tau band migrates at the known molecular weight of soluble human tau (55 kDa) and was seen only in brain samples from rTg4510 mice that overexpress human tau (*lanes labeled TG*), but not in brain samples from wild type C57BL6 mice (*lanes labeled WT*). **c** The 55 kDa TAMRA labeled band was insensitive to PNGase F treatment (PNG), required Gal-T1 treatment and was abolished by β-elimination (β-EL), thus further validating this band as O-tau

To further understand the mechanism by which OGA inhibition reduces tauopathy, we assessed whether tau was O-GlcNAcylated in rTg4510 mouse brain and then investigated the impact of OGA inhibition on O-tau levels. Although the antibody-based detection of O-tau has been described in the literature [7, 18], we were not able to detect O-tau in rTg4510 brain using anti-O-GlcNAc antibodies such as RL2 (J. Lee and G. Terracina, unpublished data) or the anti-O-tau antibody 3925 (Additional file 3). Thus, we developed a gel-based O-tau assay using “click” chemistry with the Click-iT^(R)^ labeling kit. The overall scheme of this assay is shown in Fig. 7a and is described in detail in Methods. This assay identified a specific TAMRA-labeled band with several characteristics consistent with O-tau, namely that it was immunoprecipitated by an anti-tau antibody, detected by an anti-tau antibody in Western blot, co-migrated at the known molecular weight (55 kDa) of soluble human tau, and was seen in rTg4510 mice that overexpress human tau, but not in wild type mice (Fig. 7b). The identity of this band as O-tau was further confirmed by its resistance to PNGase F, which removes only N-linked glycans, and its sensitivity to β-elimination, which removes O-linked glycans (Fig. 7c). These data thus demonstrate the successful detection of O-tau in rTg4510 mouse brain.

**Fig. 8:**
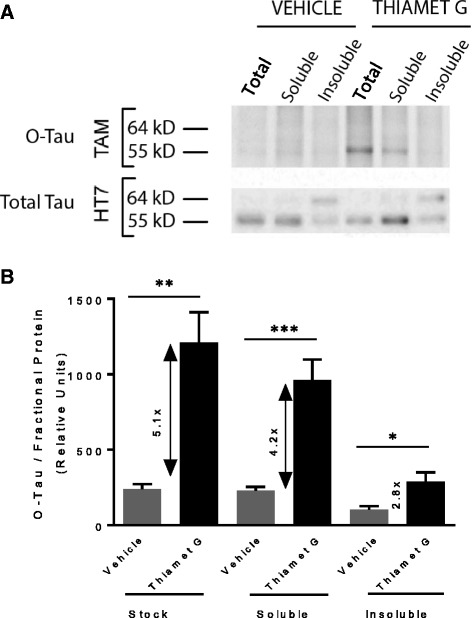
Thiamet G treatment increases O-tau levels. rTg4510 mice were treated with Thiamet G (500 mg/kg/day) for 8 weeks from 8 to 16 weeks of age. Brains were harvested at the end of study to prepare total brain homogenate, soluble and insoluble fractions. **a** Thiamet G treatment led to elevated O-tau in the total brain homogenate and soluble fractions as detected by TAMRA (TAM) labeling, but had no effect on total tau as detected by HT7 antibody on Western blots (HT7). The data shown are from one vehicle and one Thiamet G-treated animal and are representative of 5 animals in each group. **b** Quantification of O-tau normalized to total tau from the data shown in **a**. Unpaired t-test was used for comparison of vehicle and Thiamet G treatment *p* = 0.0013, 0.0008 and 0.014 for brain stock, soluble and insoluble fractions, respectively

Using this gel-based assay, we examined the effect of Thiamet G on O-tau in different rTg4510 brain fractions. O-tau was significantly elevated by 500 mg/kg/day Thiamet G treatment from 8 to 16 weeks, while total tau was unchanged (Fig. 8a). At 16 weeks of age, tau in rTg4510 mouse brain starts to shift from a 55 kDa species enriched in the soluble fraction to a hyperphosphorylated 64 kDa species that is enriched in the insoluble fraction [13]. Interestingly, O-GlcNAc was mostly associated with the soluble 55 kDa tau species, but not the insoluble, hyperphosphorylated 64 kDa tau species (Fig. 8a). Quantitation of the gels demonstrated a 4–5 fold increase of O-tau in the brain homogenate and soluble fraction and a smaller 2.8-fold increase of O-tau in the insoluble fraction after Thiamet G treatment, the latter being due primarily to the presence of some 55 kDa soluble tau in the insoluble fraction (Fig. 8b).

## Discussion

Several previous reports have suggested that OGA inhibition represents a promising treatment for neurodegenerative diseases characterized by tauopathy [7–9]. These previous studies all concluded that chronic treatment with the potent and selective OGA inhibitor Thiamet G leads to a reduction of pathological tau in transgenic mice that overexpress human tau. Some studies also demonstrated positive effects of chronic Thiamet G treatment on various behaviors and phenotypes thought to be dependent on pathological tau. The results of our study are generally in agreement with these previous findings. Indeed, chronic treatment of rTg4510 mice with 500 mg/kg Thiamet G for 8 weeks (from 8 to 16 weeks of age) led to a significant reduction of several species of tau associated with tauopathy in the brain insoluble fraction. Four weeks of chronic Thiamet G treatment (from 12 to 16 weeks of age) showed qualitatively similar, albeit non-significant, effects. This is consistent with previous studies showing a progressive increase in the effects of OGA inhibition with increased treatment time [8, 9]. Alternatively, the reduced effect may be due to the fact that tauopathy is more advanced between 12 and 16 weeks of age and thus less amenable to OGA inhibitor treatment. The 500 mg/kg dose of Thiamet G was chosen because both genetic knockdown and preliminary pharmacological studies suggested that virtually complete inhibition of OGA is required to significantly increase brain O-protein levels in the rTg4510 mouse model. As expected, this dose of Thiamet G achieved complete inhibition of brain OGA and resulted in a ~ 5-fold increase in brain O-protein levels as assessed by a sensitive, high throughput assay developed for this study. The novel finding that virtually complete inhibition of OGA is required for a measurable pharmacodynamic effect and the development of a sensitive, high throughput assay for measurement of O-protein levels will greatly facilitate dose selection and quantitation of OGA inhibition in human clinical trials. The high degree of OGA inhibition required to detectably change brain O-protein levels suggests that OGA activity is tightly regulated to ensure the homeostasis of protein O-GlcNAcylation. Regulatory processes that compensate for the effects of OGA inhibition include substrate accumulation and an increase of OGA expression (Additional file 4); in contrast, no change in OGT levels was observed (data not shown). No overt phenotypes were observed either in the inducible OGA knockdown mice or after chronic treatment with Thiamet G, providing initial evidence that substantial OGA inhibition is well tolerated.

Although there is a consensus that OGA inhibition reduces pathological tau, the mechanism by which OGA inhibitors achieve this effect is unclear, and this uncertainty has hindered the advancement of OGA inhibitors for the treatment of tauopathies [10]. One proposal is that tau is not normally modified by O-GlcNAc and that OGA inhibition influences tau pathology via an indirect mechanism, e.g. by altering O-GlcNAcylation of proteins other than tau itself [8]. The data in the present study do not support this proposal. By employing a novel O-GlcNAc detection method involving “click” GalNAz derivatization coupled with TAMRA labeling, we clearly demonstrated O-GlcNAc modification of tau after chronic treatment of rTg4510 mice with Thiamet G. Our results are consistent with previous studies that used O-tau specific antibodies to demonstrate O-GlcNAcylation of tau and elevation of O-tau upon Thiamet G treatment [7, 9]. The conclusion that tau is not directly O-GlcNAcylated was based on an inability to measure O-tau with antibody 3925 [8]. In our hands, antibody 3925 can detect high levels of O-tau (e.g. when HEK293 cells are co-transfected with tau and OGT and treated with Thiamet G), but does not have sufficient sensitivity to detect the low levels of O-tau in brain homogenates from rTg4510 mice under normal conditions or after treatment with Thiamet G (see Additional file 3). While further work is required to clarify the conflicting results with antibody 3925 [7, 19], the independent detection of O-tau by click chemistry labeling suggests that an increase in O-GlcNAcylation of tau is the most parsimonious explanation for the ability of OGA inhibition to reduce tau pathology in rTg4510 mice. As concluded previously, however [8], we cannot completely rule out the possibility that increased O-GlcNAcylation of proteins other than tau may also contribute to the efficacy of Thiamet G.

A reciprocal relationship between O-GlcNAcylation and phosphorylation of proteins has commonly been cited as the mechanism for OGA inhibitor effects [5, 15]. Previous studies have suggested that OGA inhibition (and the presumed increase in tau O-GlcNAcylation) reduces tau phosphorylation after acute but not chronic treatment [7, 15, 20]; reduces tau phosphorylation [9] or total tau [7, 20] only in the pathological 64 kDa, sarkosyl-insoluble fraction; or bi-directionally alters the levels of various phosphorylated forms of tau [21]. The present study does in fact demonstrate reduction of several phosphorylated tau species as well as global tau phosphorylation in the insoluble fraction of brain homogenates after chronic Thiamet G treatment. However, several observations do not support a reciprocal interplay between O-GlcNAcylation and phosphorylation of specific amino acid residues of tau as the mechanism by which OGA inhibition reduces tau pathology. OGA inhibition reduces phosphorylation of the insoluble 64 kDa tau species, but O-GlcNAcylated tau was detected primarily in the 55 kDa tau species (present study, [9, 15, 22]). In addition, several phosphorylated species of tau (PHF6 tau, pThr tau, pT181 tau, pS202/pT205 tau and pT231 tau) were reduced in the insoluble fraction of brain after OGA inhibition, and the levels of these phosphorylated tau species were highly correlated across all animals and treatment groups. These results are consistent with our previous finding that many post-translationally modified forms of tau are highly correlated with one another throughout the lifespan of rTg4510 mice [13] and may suggest that OGA inhibition has a widespread rather than a discrete effect on phosphorylation and other post-translational modifications of tau (see below). Finally, it has recently been shown that very few of the known tau phosphorylation sites are modified by O-GlcNAc, either under normal physiological conditions [19] or after treatment of rTg4510 mice with Thiamet G [23] Even when tau and OGT are overexpressed in *E. coli*, only ~1.5 O-GlcNAc residues are detected per tau molecule [24]. Nevertheless, it remains formally possible that OGlcNAcylation reciprocally reduces tau phosphorylation at one or a very few specific sites that are critical in driving tau into an insoluble, aggregated, pathological state, and that these O-GlcNAc modifications are thus present at low and undetectable levels in the insoluble fraction.

It has also been proposed that the addition of polar O-GlcNAc moieties to tau reduces the propensity of tau to aggregate, as shown in in vitro experiments [7, 22]. O-GlcNAcylation has also been shown to reduce the aggregation propensity of other proteins such as α-synuclein and polyhomeotic [25, 26]. However, these in vitro aggregation studies used recombinant or synthetic proteins, which may have distinct sites and stoichiometry of O-GlcNAc modification compared to the native proteins in vivo. The present study demonstrates that chronic OGA inhibition reduces tau aggregates in the brains of rTg4510 mice, thus adding in vivo validation to the in vitro results. In agreement with recent results obtained with a novel O-tau antibody [9], we also found that O-GlcNAc was primarily associated with the soluble 55 kDa tau species, but not the insoluble 64 kD pathological tau species. This argues that O-GlcNAc modification maintains tau in a non-pathological, unaggregated state with limited post-translational modifications (55 kDa) and prevents the formation of insoluble, aggregated, highly modified pathological tau (64 kD). While reduction of the aggregation propensity of tau is the most plausible explanation, additional work is necessary to better understand the molecular mechanism by which OGA inhibition reduces formation of pathological tau.

The varying conclusions reached in different studies regarding the potential mechanisms by which OGA inhibition reduces tau pathology may largely be attributed to the different methods and readouts used [7–9, 15, 24]. Different transgenic mouse models have been used to test the effects of OGA inhibition, and there is significant variability in age-related tau pathology development within and between the various animal models. We compensated for this variability by using a large number of female animals per treatment group to sufficiently power our analysis [13]. In addition, the high throughput immunoassays employed in this study are much more robust in detecting modest effects of OGA inhibition on various tau species than the Western blot methods used in other studies. Furthermore, the brain homogenate used in many studies in which p-tau species were measured [7, 20] is a more heterogeneous pool of different tau species than the soluble and insoluble brain fractions we used here [7, 13, 20].

It has long been known that CSF total tau and p-tau are elevated in AD patients compared to age-matched controls [27]. In rTg4510 mice, CSF total tau also displays age-dependent changes during the accumulation of pathological tau in the brain [12, 13]. This study reports for the first time an effect of OGA inhibitor treatment on CSF tau levels. Similar to the reduction of pathological species of tau in the brain, we observed a significant reduction of CSF total tau when rTg4510 mice were treated for 8 weeks with Thiamet G. In a shorter 4 week treatment paradigm, Thiamet G administration did not alter CSF total tau, again consistent with the less robust reduction in pathological tau in the brain achieved in this time frame. It is also interesting to note the strong correlation between CSF total tau and pT181 tau in response to OGA inhibition. Even though the reduction of pT181 tau did not reach statistical significance, the correlation between CSF total tau and pT181 tau suggests that these two endpoints reflect effects of OGA inhibition on the same pool of tau in CSF. Therefore, CSF total tau may serve as an important translational biomarker for OGA inhibitor development.

The results of this study clarify the mechanism by which OGA inhibition reduces pathological tau and describes translational knowledge and tools that will facilitate human clinical development of OGA inhibitors. The treatment paradigm (i.e. treatment from 8 to 16 weeks of age) was optimized to maximize the window to observe effects of OGA inhibition, allow detection of the earliest stages of pathological tau development, and to assess treatment effects at these early stages of pathology, a time frame in which efficacy of OGA inhibition (or any tau-based therapy) is likely to be most effective. Neurofibrillary tangle pathology and neurodegeneration in rTg4510 mice are not well-developed by 16 weeks of age [14], thus providing minimal or no window to observe effects of Thiamet G on these parameters. In a study with the OGA inhibitors MK-8719 and Thiamet G to be published separately, reduction of neurofibrillary tangle pathology and neurodegeneration were observed after treatment between 8 and 20 weeks of age [28]. Furthermore, the 64 kDa insoluble tau species measured in this study has been shown to correlate well with neurofibrillary tangle pathology [14].

We have previously shown that tauopathy in rTg4510 mice results from a more or less simultaneous repertoire of events, including conversion of tau from a soluble 55 kDa species to an insoluble 64 kDa species, multiple post-translational modifications (e.g. phosphorylation, acetylation, nitration, ubiquitination), impaired proteostasis, aggregation and NFT formation [13]. A corollary of this notion is that an ideal treatment for tauopathy should lead to a reduction of all these features, and indeed OGA inhibition appears to fulfill this criterion. Ongoing clinical trials with OGA inhibitors (e.g. [29]) will provide additional insight into the potential of this mechanism to reduce tauopathy.

## Conclusion

The present study demonstrates that nearly complete inhibition of OGA via chronic treatment with Thiamet G reduces both pathological tau in the brain and total tau in the CSF of rTg4510 mice, thereby providing additional evidence that OGA inhibition may represent a safe and effective treatment for Alzheimer’s disease and other neurodegenerative diseases characterized by tauopathy. We have demonstrated that tau itself is O-GlcNAcylated and that OGA inhibition most likely reduces pathological tau via a direct effect to increase O-tau and maintain tau in a soluble, unaggregated, non-pathogenic form. This added clarity around the mechanism by which OGA inhibition reduces tauopathy, the demonstration that nearly complete inhibition of OGA is required to achieve a measurable pharmacodynamic effect (increase in O-protein), the development of sensitive high throughput assays for detection of this pharmacodynamic effect and the identification of CSF total tau as a potential translational biomarker will support the clinical development of OGA inhibitors.

## Methods

### Reagents

Thiamet G was synthesized as described [15]. Tritiated Thiamet G was prepared from intermediate **1** by the scheme outlined below [30]. Reduction of the aldehyde of intermediate **1** using sodium borohydride[^3^H] followed by deprotection and purification gave [^3^H]Thiamet G in high chemical yield with a specific activity of 16.6 Ci/mmole.
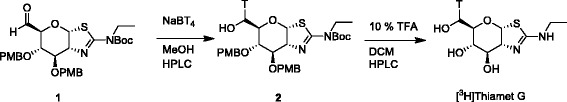


The sources and epitopes of antibodies used in this study have been described in detail previously [13].

### Cell culture

HEK293 cells were obtained from ATCC and cultured under standard conditions in Dulbecco’s modified Eagle’s media supplemented with 10% fetal bovine serum. The cells were treated for 6 h with vehicle or Thiamet G in 1% DMSO, followed by lysing and harvesting in a buffer containing 50 mM Tris (pH 8.0), 250 mM NaCl, 5 mM KCl, 2 mM EDTA, 2 mM EGTA, 0.2% Tween 20, 1 μM PUGNAC (Sigma-Aldrich) and 1 tablet of EDTA-free protease inhibitor cocktail (Roche) per 10 mL of buffer.

### Animal experiments

All animal studies were conducted in an AAALAC- accredited facility in accordance with policies of the Merck Institutional Animal Care and Use Committee and the National Research Council’s Guide for the Care and Use of Laboratory Animals.

### rTg4510 mouse studies

rTg4510 mice were bred by crossing the hTau responder line (FVB/N strain) and the tTA activator line (129S6 strain) as described previously [11]. In our previous study, we found a significant difference in the age-dependent development of tau pathology between male and female rTg4510 mice [13]. To minimize this source of variability, only F1 female rTg4510 mice (FVB/129S6 strain) were used [13] and the study was designed with 80 + % power to detect a 30% effect of Thiamet G administered between 8 and 16 weeks of age and 50–60% power to detect a 30% effect of Thiamet G administered between 12 and 16 weeks of age. For the chronic efficacy studies, Thiamet G was added at a concentration of 3.3 mg/g to normal mouse chow (D01060501, Research Diets, Inc., New Brunswick, NJ) to achieve in-diet dosing of ~500 mg/kg/day. The normal mouse chow without added Thiamet G was used as the control diet. The control diet was provided ad libitum to all animals beginning at 6 weeks of age. For Thiamet G-treated groups (*n* = 35 per group), the diet was switched to chow containing Thiamet G at either 8 weeks of age (8 weeks of dosing) or 12 weeks of age (4 weeks of dosing). The vehicle-treated group (*n* = 35 per group) received control diet throughout the study. All animals were euthanized at 16 weeks of age. In addition, two baseline groups that were not treated with Thiamet G were euthanized at 8 and 12 weeks of age (*n* = 10 per group). Body weight and food consumption were measured weekly during the study.

### Preparation of homogenates, soluble fraction and insoluble fraction from brain

The brain homogenates and the soluble and insoluble brain fractions were prepared as described previously [13] with some modifications. Briefly, the brain homogenates were prepared by homogenizing each brain hemisphere in 900 μL of ice cold buffer comprised of 50 mM Tris (pH 8.0), 250 mM NaCl, 5 mM KCl, 2 mM EDTA, 2 mM EGTA, 1 μM PUGNAC (Sigma-Aldrich) and 1 tablet each of Phos-Stop (Roche) and EDTA-free protease inhibitor cocktail (Roche) per 10 mL of buffer. The brain homogenate was then centrifuged at 15,000 g for 15 min at 4 °C, and the resulting supernatant (designated in this study as total brain homogenate) was centrifuged at 100,000 g for 30 min at 4 °C. The supernatant and pellet from this high speed spin are designated in this study as the soluble and insoluble fraction, respectively.

### O-protein assay

A sandwich immunoassay was used to assess total O-protein. Five μg/mL biotinylated wheat germ agglutinin (WGA, Vector Biology) in 50 μL phosphate-buffered saline (PBS) containing 5% Blocker A (MSD) and 0.2% Tween 20 was used to coat 96-well avidin plates (MSD). After incubation for 1 h at room temperature, the plates were washed three times with 0.2% Tween 20 in PBS. Twenty five μL of total brain homogenate or cell lysate (~100 μg protein) were then added and the plates were incubated for 3 hours at room temperature before being washed three times with 0.2% Tween 20 in PBS. This procedure captures glycosylated proteins containing terminal N-acetylglucosamine modification [31, 32]. Subsequently, an antibody that recognizes O-GlcNAc moieties on the captured proteins (RL2, 1:1000 dilution, Abcam) [33] and S-tagged goat anti-mouse antibody (MSD) were added in 50 μL of 5% Blocker A and 0.2% Tween 20 and the plates were incubated at 4 °C overnight. The plates were then washed three times with Wash Buffer (MSD), 150 μL Read Buffer T (MSD) was added, and the plates were analyzed on Sector 6000 Imager (MSD).

### OGA equilibrium binding assay

On the day binding assays were performed, the brain homogenates (prepared as described above) were thawed on ice and adjusted to a protein concentration of 0.6 μg/uL with binding assay buffer (BAB; 20 mM HEPES (pH 7.4), 100 mM NaCl, 3 mM MgCl_2_, 0.1% BSA). Assay reactions (500 μL final volume) were assembled at room temperature in 96 deep-well assay blocks (2 mL volume/well) by combining 180 μL BAB, 20 uL BAB with or without 20 μM unlabeled Thiamet-G (to determine non-specific binding), and 50 uL BAB containing 0–300 nM [^3^H]Thiamet G. The reactions were then initiated by adding 250 μL of brain homogenate and incubated at room temperature for 4 h with gentle agitation. The reactions were terminated by aspiration via a Brandel tissue harvester onto Unifilter GF/B Whatman filter plates pre-soaked for 30 min in ice cold 50 mM Tris (pH 7.4), 0.3% *v*/v polyethyleneimine. The plates were rapidly washed with ice-cold 5 mM Tris-Cl (pH 7.4) before being dried and the plate bottoms sealed. Radioactivity bound to the filter plate was counted on a TopCount-NXT scintillation spectrophotometer (PerkinElmer) after adding 60–70 μL Microscint-20 (Perkin Elmer) per well. K_d_ and B_max_ values for [^3^H]Thiamet G binding to OGA in brain were determined by averaging replicates of total and non-specific binding and simultaneously fitting the mean values to equations describing these parameters [34].

### O-tau assay

The total brain homogenates, as well as the soluble and insoluble fractions from rTg4510 or C57BL6 mouse brain, were prepared as described above. O-GlcNAcylated tau protein (O-tau) in these brain samples was determined as shown in Fig. 7a. For removal of N- and/or O-linked glycans, mouse brain samples (300 μg protein) were precipitated by standard methanol/chloroform extraction [35] and re-suspended in 30 μL of buffer containing 1% SDS and 20 mM HEPES (pH 7.9). Sample volumes were brought to 240 μl with 20 mM HEPES, (pH 7.9) (without SDS) and 30 μL of a denaturing solution containing 1% octyl-beta-D-glycopyranoside/0.005% 2-mercaptoethanol (both from Sigma-Aldrich) were added. The samples were then boiled for 5 min and rapidly cooled on ice. A final 30 μL of a solution of 10% Triton-X100 in 20 mM HEPES (pH 8) was added to neutralize the remaining SDS, followed by addition of 5 units of the glycoamidase PNGase F (Sigma-Aldrich) to remove N-linked glycans [36]. The reactions were incubated at either 37 °C for 3 h or at 4 °C overnight. In some cases, O-linked glycans were also removed after precipitating the completed PNGase F reaction and resuspending the proteins in water, followed by alkali-mediated β-elimination using a commercially available kit (Sigma-Aldrich). The β-elimination reaction proceeded overnight at 4 °C, after which the reactions were carefully neutralized using 1 M HCl.

Following the removal of N- and/or O-linked glycans, samples were re-precipitated with methanol-chloroform as described above and solubilized in 50 μL of 1% SDS/50 mM Tris-Cl (pH 8.0). The terminal N-acetyl-beta-D-glucosamine (GlcNAc) residues of glycoproteins present in mouse brain samples were labeled with N-azido-galactose using a Click-IT kit according to the manufacturer’s instructions (Invitrogen catalog #33368). Alkyne-conjugated tetramethylrhodamine (TAMRA) was added to the resulting azide by means of copper-catalyzed Huisgen cycloaddition using a commercial kit (Invitrogen). After assembling the reactions as directed by the kit protocol, the reaction tubes were protected from light and rotated at room temperature for 20 min. The reactions were then precipitated with methanol-chloroform as described above and the pellets were solubilized in 75 μL of homogenization buffer.

TAMRA-labeled tau protein was immunoprecipitated using biotinylated anti-human tau antibody HT7 (ThermoFisher) followed by capture with streptavidin-conjugated magnetic Dynabeads (Invitrogen). Captured TAMRA-labeled tau was then eluted from the beads into 30 μL homogenization buffer by heating the samples at 55 °C for 5 min. The eluted proteins, along with Odyssey infrared size markers (Li-Cor Biosciences), were separated on 12% Bis-Tris polyacrylamide gels (NuPage Novex) in 1× MOPS running buffer. TAMRA labeled O-tau was detected in the gels using a Typhoon Model FLA9000 laser biomolecular imager (excitation 542 nm) and Fujifilm LPG emission filter (GE). Data were captured and analyzed using ImageQuant TL software. Following imaging, the gel was transferred onto nitrocellulose membranes and Western blot analysis was performed using the HT7 antibody (1:1000 dilution, ThermoFisher) and ECL anti-mouse secondary antibody (1:2000 dilution, GE Healthcare). The infrared fluorescence of the size markers and chemiluminescence of the samples were captured using an Odyssey Fc Bioimager (Li-Cor Biosciences) at 700 nm and 480 nm, respectively, and quantified using ImageQuant TL software.

### Brain tau assays

Total tau, p-tau and tau aggregates were measured using sandwich immunoassays on the AlphaLISA platform (Perkin Elmer) as described previously [13].

### CSF tau assays

CSF total tau was measured as described [13] using an AlphaLISA assay with biotinylated HT7 antibody and BT2 antibody (both from ThermoFisher) conjugated to acceptor beads (PerkinElmer). CSF pT181 tau was measured using an immunoassay on the Erenna platform (Singulex) with HT7 as the capture antibody and AT270 as the detection antibody. Assays measuring CSF pT181 tau were carried out in the presence of Halt^R^ Protease and Phosphatase Inhibitor Cocktail (Thermo Fisher). Assays (100 μl) were carried out in 96-well format with each well containing 10 mg capture beads in 50 μl Singulex assay buffer plus 50 μl standard peptide or sample (2 μl CSF from rTg4510) diluted in 0.2% Tween 20 in PBS. Following incubation at 25 °C for 4 h, the plates were washed according to the manufacturer’s instructions using the Biotek-405 Select TS. Twenty μl of AT270 antibody (50 ng/mL) was then added and the plate was incubated overnight at 25 °C with shaking. The plate was developed according to the manufacturer’s instructions and data analysis was conducted using Sgx link software (Singulex). A standard curve was established using the synthetic peptide APPGQKGQA—TTDS-TTDS-TTDS-TTDS—YS-pSer-PG-pSer-PG-pThr-PGSRS (JPT Peptide Technologies), which incorporates sequences recognized by HT7 and AT270 linked together with four molecules of ((N-(3-[2-[2-(3-Amino-propoxy)-ethoxy]-ethoxy]-propyl)-succinamic acid) (TTDS; CAS number 723312–72-9).

### Determination of Thiamet G concentrations in brain and plasma

Brains were homogenized in water at a 1:3 weight/volume ratio using a GenoGrinder homogenizer. Proteins in plasma samples and brain homogenates were precipitated using acetonitrile containing an internal standard. Untreated brain and plasma were used to prepare standards and quality control samples to cover the expected range of Thiamet G concentrations. After protein precipitation, samples were centrifuged at 5000 g and the supernatants were collected for LC-MS analysis using a Transcend System HPLC connected to an API 5000 triple quadrupole mass spectrometer (Thermo Scientific).

### Statistical analysis

Correlation and t-test analyses were conducted using Prism 5.0 software (GraphPad). Two-way repeated measures ANOVA followed by Bonferroni post hoc test was used for body weight and food intake analysis.

## Additional files

Additional file 1: Generation of OGA iKD mice [36, 37]. (DOCX 117 kb)

Additional file 2: Analysis of OGA mRNA expression in OGA iKD and littermate WT mice after treatment for 10 days with or without doxycycline. shRNA-mediated knock-down of OGA was achieved by in-diet treatment with doxycycline for 10 days as described in Additional file 1, after which animals (*n* = 3 per group) were sacrificed and various tissues were harvested. The RNeasy Lipid Tissue Mini Kit (Qiagen) was used for RNA isolation from tissues according to the manufacturer’s instructions. Pre-configured TaqMan Gene Expression Assays (Applied Biosystems) were used to assess the OGA mRNA level with the Hp1bp3 housekeeping gene as the normalization control. To compare OGA mRNA expression levels between treatment and genotype, the CT values for WT mice without doxycycline treatment were set at 100% and CT values for the other treatment/genotype groups were expressed relative to that value. Absolute CT values for each tissue were: brain, 24; aorta, 26; heart, 25; muscle, 25; SI, 25; colon, 24; EWAT, 26; kidney, 23; liver, 25; pancreas, 30. OGA mRNA expression was detected in all tissues examined, with particularly high expression levels in brain, colon, and kidney. About 70% knock-down of OGA mRNA levels was achieved in brain in the OGA iKD mice treated with doxycycline, but a 35% knock-down of OGA mRNA was also noted for OGA iKD mice that were fed diet without doxycycline, indicating some leakiness in the expression of the shRNA construct in the brain. No other tissue showed evidence of leaky expression of the shRNA construct in the absence of doxycycline treatment. Substantial knock-down of OGA mRNA expression was observed in all tissues in response to doxycycline treatment, with the lowest amount of OGA mRNA remaining in the heart (12%), kidney (18%) and liver (25%). Abbreviations: SI, small intestine; EWAT, epididymal white adipose tissue. (PPTX 99 kb)

Additional file 3: Detection of O-tau in HEK293 cell lysates and in mouse brain homogenates using antibody 3925. A. Western blot analysis of O-tau in HEK293 cells. HEK293 cells were transiently transfected with pcDNA3.1 vector alone, with pcDNA3.1 vector containing human 2N4R tau cDNA, or with pcDNA3.1 vectors containing human 2N4R tau cDNA and human OGT. Three days after transfection, cells were treated overnight with 1 μM Thiamet G or vehicle (water). Cells were then lysed as described in Methods, proteins (10 μg) were separated by SDS-PAGE and transferred to nitrocellulose, and the nitrocellulose membranes were probed with antibody 3925 (kindly provided by Dr. David Vocadlo at Simon Fraser University, Burnaby, Canada) at a dilution of 1:500 [7]. Purified O-tau from Sf9 cells was also loaded as a control. Antibody 3925 detected O-tau in HEK293 cells only when tau and OGT were co-expressed and the cells were treated with Thiamet G. The antibody also recognized a non-specific protein with similar molecular weight to tau that was present in the vector transfected cells and was not affected by either OGT expression or Thiamet G treatment. B. Western blot analysis of total brain homogenates from wild-type and rTg4510 mice treated with vehicle, 12.5 mg/kg or 125 mg/kg Thiamet G for 7 days. Antibody 3925 did not detect a protein having the molecular weight of O-tau, but did detect a non-specific protein with molecular weight of 37.5 kD that was also present in the brain homogenate from tau knockout mice. (PPTX 247 kb)

Additional file 4: Elevation of OGA expression following Thiamet G treatment. rTg4510 mice at 8 weeks of age were treated with vehicle or 100 mg/kg Thiamet G formulated in diet for 12 weeks (*n* = 25 per group). The brain tissues were analyzed for OGA mRNA level (A) or OGA protein expression using an anti-OGA antibody (Santa Cruz Biotechnology) (B). Both mRNA and protein were elevated ~2-fold following chronic treatment with Thiamet G. (PPTX 150 kb)

## Abbreviations

AD: Alzheimer’s disease
BAB: Binding assay buffer
CSF: Cerebrospinal fluid
ES cells: Embryonic stem cells
iKD: Inducible knockdown
MSD: MesoScale discovery
NFT: Neurofibrillary tangle
OGA: O-GlcNAcGlcNAcase
O-protein: O-GlcNAc modified protein
O-tau: O-GlcNAcGlcNAc modified tau
PBS: Phosphate-buffered saline
PHF: Paired helical filament
PSP: Progressive supranuclear palsy
p-tau: Phosphorylated tau
RCME: Recombinase-mediated cassette exchange
SEM: Standard error of the mean
TAMRA: Tetramethylrhodamine
TBS: Tris-buffered saline
WGA: Wheat germ agglutinin
WT: Wild type
