# Supplementary material for: Inhibition of O-GlcNAcase leads to elevation of O-GlcNAc tau and reduction of tauopathy and cerebrospinal fluid tau in rTg4510 mice
Source: Mol Neurodegener. 2017 May 18;12:39. doi: 10.1186/s13024-017-0181-0 (PMC5437664; doi:10.1186/s13024-017-0181-0)
Supplement: Supplementary file 1 — Generation of OGA iKD mice [36, 37]. (DOCX 117 kb) [file 13024_2017_181_MOESM1_ESM.docx]

**Supplement 1. Generation of OGA iKD mice.**

OGA (official gene symbol: Mgea5) iKD mice were generated at TaconicArtemis (Cologne, Germany) and subsequently bred at Taconic Biosciences Inc. (Germantown, NY). The mice were generated by recombinase-mediated cassette exchange (RMCE) as described previously [36, 37]. The shRNA sequence directed against OGA (Mgea5) mRNA (5'- GCAAACAGCAGTGTTGTCA-3') was cloned into the pRMCE-H1 vector. Targeted C57BL6 ES cells were injected into blastocysts and chimeras were bred to C57BL/6NTac mice for germ line transmission. Only one allele of the *Rosa*26 locus is targeted in the OGA iKD mice, which were bred with C57BL/6NTac mice to generate heterozygous iKD and wild-type (WT) littermate control mice. OGA iKD mice were genotyped by PCR using the following conditions: 95°C for 5 min; followed by 35 cycles of 95°C for 30 sec, 60°C for 30 sec and 72°C for 1 min; followed by 72°C for 10 min. The PCR primer pairs used for genotyping were 5’-CCATGGAATTCGAACGCTGACGTC-3’ (forward) and 5’- TATGGGCTATGAACTAATGACCC-3’ (reverse), which amplifies a 381 bp fragment of the vector including the shRNA cassette; and 5’-GAGACTCTGGCTACTCATCC-3’ (forward) and 5’- CCTTCAGCAAGAGCTGGGGAC-3’ (reverse), which amplifies a 585 bp fragment from the wild-type allele as a control. To induce shRNA-mediated knock-down of OGA, OGA iKD mice and WT littermate controls were fed a doxycycline-containing diet for 10 days (1 g/kg doxycycline; ssniff special diets, Soest, Germany, S8289-P012, www.ssniff.de). As a control, mice were fed a matching diet without doxycycline (ssniff special diets, V1154-3).
