# Supplementary figures and images for: Inhibition of O-GlcNAcase leads to elevation of O-GlcNAc tau and reduction of tauopathy and cerebrospinal fluid tau in rTg4510 mice
Source: Mol Neurodegener. 2017 May 18;12:39. doi: 10.1186/s13024-017-0181-0 (PMC5437664; doi:10.1186/s13024-017-0181-0)

## Slide 1
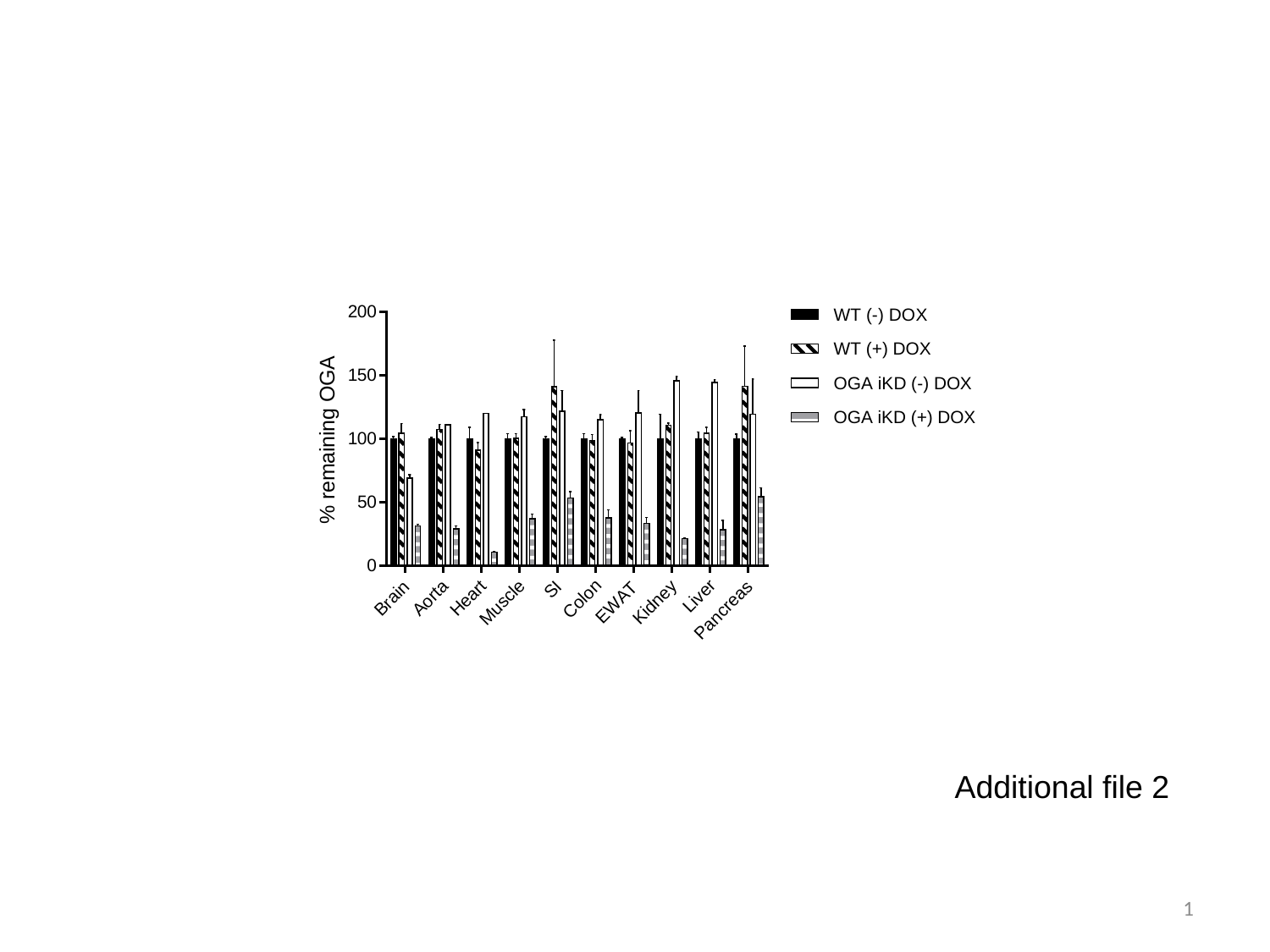

Additional file 2
1

Supplement: Supplementary file 2 — Analysis of OGA mRNA expression in OGA iKD and littermate WT mice after treatment for 10 days with or without doxycycline. shRNA-mediated knock-down of OGA was achieved by in-diet treatment with doxycycline for 10 days as described in Additional file 1, after which animals (n = 3 per group) were sacrificed and various tissues were harvested. The RNeasy Lipid Tissue Mini Kit (Qiagen) was used for RNA isolation from tissues according to the manufacturer’s instructions. Pre-configured TaqMan Gene Expression Assays (Applied Biosystems) were used to assess the OGA mRNA level with the Hp1bp3 housekeeping gene as the normalization control. To compare OGA mRNA expression levels between treatment and genotype, the CT values for WT mice without doxycycline treatment were set at 100% and CT values for the other treatment/genotype groups were expressed relative to that value. Absolute CT values for each tissue were: brain, 24; aorta, 26; heart, 25; muscle, 25; SI, 25; colon, 24; EWAT, 26; kidney, 23; liver, 25; pancreas, 30. OGA mRNA expression was detected in all tissues examined, with particularly high expression levels in brain, colon, and kidney. About 70% knock-down of OGA mRNA levels was achieved in brain in the OGA iKD mice treated with doxycycline, but a 35% knock-down of OGA mRNA was also noted for OGA iKD mice that were fed diet without doxycycline, indicating some leakiness in the expression of the shRNA construct in the brain. No other tissue showed evidence of leaky expression of the shRNA construct in the absence of doxycycline treatment. Substantial knock-down of OGA mRNA expression was observed in all tissues in response to doxycycline treatment, with the lowest amount of OGA mRNA remaining in the heart (12%), kidney (18%) and liver (25%). Abbreviations: SI, small intestine; EWAT, epididymal white adipose tissue. (PPTX 99 kb) [file 13024_2017_181_MOESM2_ESM.pptx]

## Slide 1
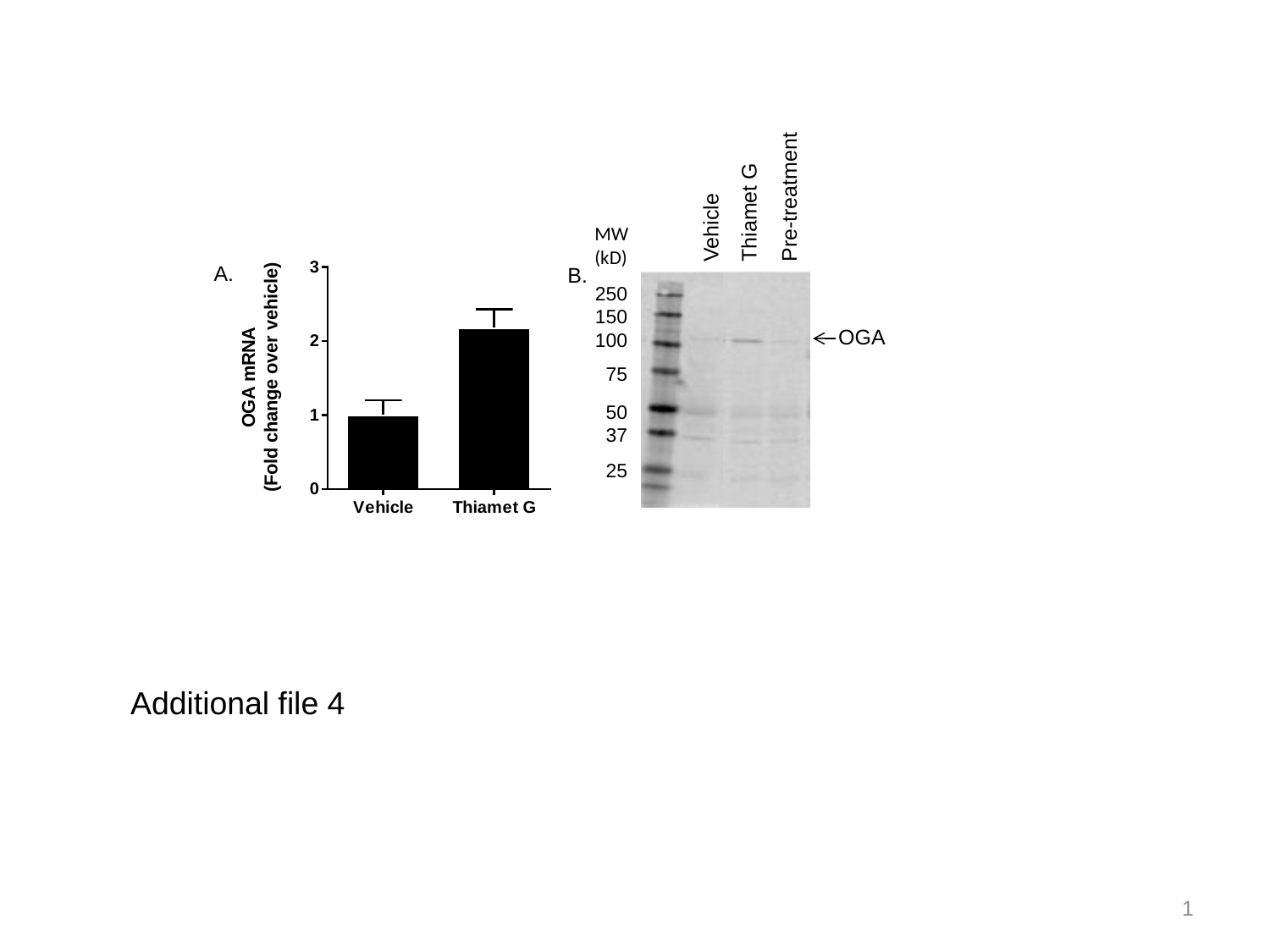

Vehicle
Thiamet G
Pre-treatment
250
150
100
75
50
37
25
OGA
MW (kD)
A.
B.
Additional file 4
1

Supplement: Supplementary file 4 — Elevation of OGA expression following Thiamet G treatment. rTg4510 mice at 8 weeks of age were treated with vehicle or 100 mg/kg Thiamet G formulated in diet for 12 weeks (n = 25 per group). The brain tissues were analyzed for OGA mRNA level (A) or OGA protein expression using an anti-OGA antibody (Santa Cruz Biotechnology) (B). Both mRNA and protein were elevated ~2-fold following chronic treatment with Thiamet G. (PPTX 150 kb) [file 13024_2017_181_MOESM4_ESM.pptx]
