# Supplementary material for: Inhibition of O-GlcNAcase leads to elevation of O-GlcNAc tau and reduction of tauopathy and cerebrospinal fluid tau in rTg4510 mice
Source: Mol Neurodegener. 2017 May 18;12:39. doi: 10.1186/s13024-017-0181-0 (PMC5437664; doi:10.1186/s13024-017-0181-0)
Supplement: Supplementary file 3 — Detection of O-tau in HEK293 cell lysates and in mouse brain homogenates using antibody 3925. A. Western blot analysis of O-tau in HEK293 cells. HEK293 cells were transiently transfected with pcDNA3.1 vector alone, with pcDNA3.1 vector containing human 2N4R tau cDNA, or with pcDNA3.1 vectors containing human 2N4R tau cDNA and human OGT. Three days after transfection, cells were treated overnight with 1 μM Thiamet G or vehicle (water). Cells were then lysed as described in Methods, proteins (10 μg) were separated by SDS-PAGE and transferred to nitrocellulose, and the nitrocellulose membranes were probed with antibody 3925 (kindly provided by Dr. David Vocadlo at Simon Fraser University, Burnaby, Canada) at a dilution of 1:500 [7]. Purified O-tau from Sf9 cells was also loaded as a control. Antibody 3925 detected O-tau in HEK293 cells only when tau and OGT were co-expressed and the cells were treated with Thiamet G. The antibody also recognized a non-specific protein with similar molecular weight to tau that was present in the vector transfected cells and was not affected by either OGT expression or Thiamet G treatment. B. Western blot analysis of total brain homogenates from wild-type and rTg4510 mice treated with vehicle, 12.5 mg/kg or 125 mg/kg Thiamet G for 7 days. Antibody 3925 did not detect a protein having the molecular weight of O-tau, but did detect a non-specific protein with molecular weight of 37.5 kD that was also present in the brain homogenate from tau knockout mice. (PPTX 247 kb) [file 13024_2017_181_MOESM3_ESM.pptx]

## Slide 1
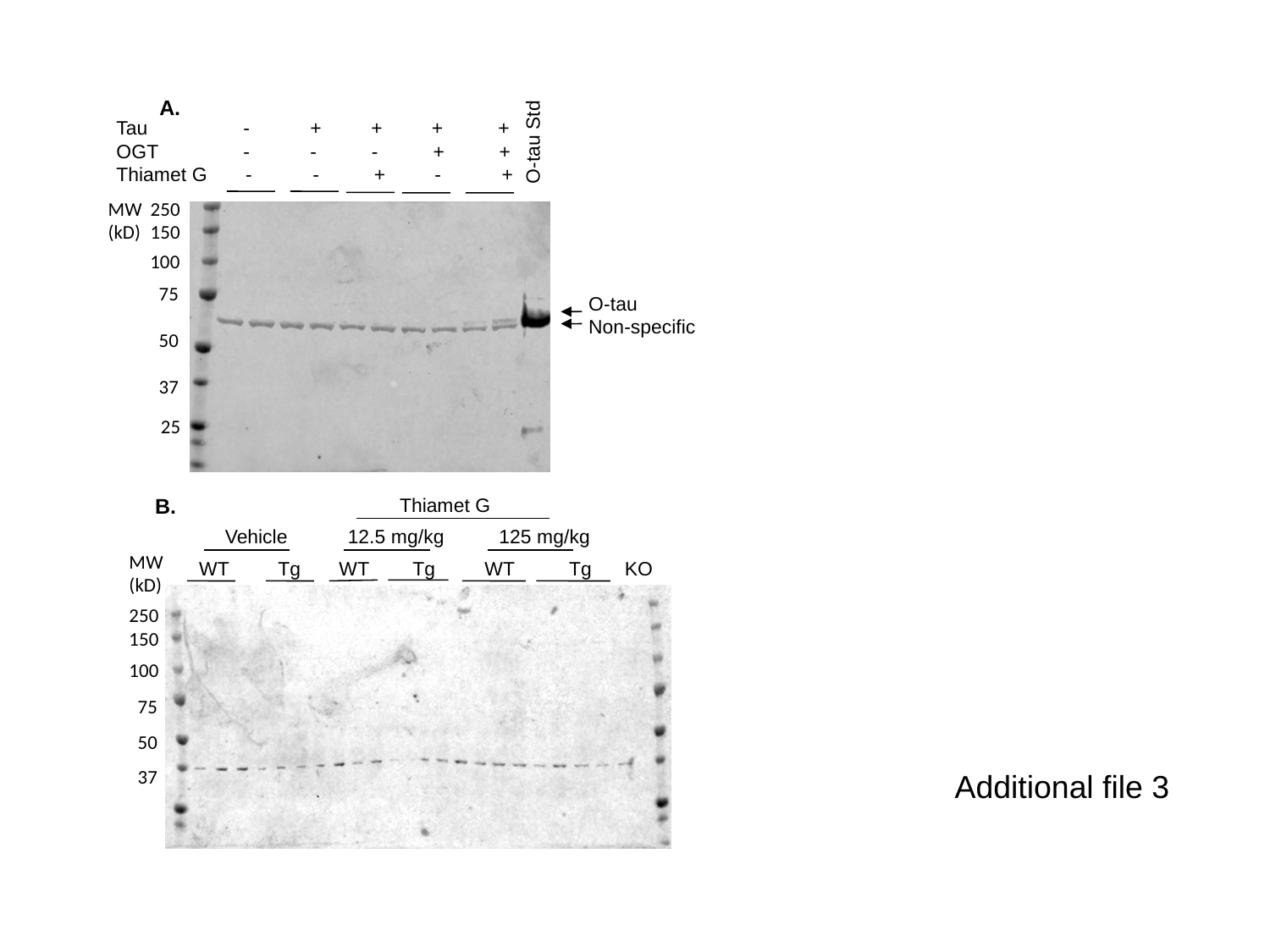

A.
O-tau Std
Tau 	- + + + +
OGT	- - - + +
Thiamet G - - + - +
O-tau
Non-specific
MW
(kD)
250
150
100
 75
 50
 37
25
B.
Thiamet G
Vehicle 12.5 mg/kg 125 mg/kg
WT Tg WT Tg WT Tg KO
MW
(kD)
250
150
100
 75
 50
 37
Additional file 3
